# Updates on laparoscopic cervical cerclage: obstetric outcomes and surgical techniques

**DOI:** 10.2144/fsoa-2023-0051

**Published:** 2023-07-17

**Authors:** Wael Abdallah

**Affiliations:** 1 Department of Gynecology & Obstetrics, Hôtel-Dieu de France University Hospital, Saint Joseph University, Beirut, Lebanon

**Keywords:** cervical cerclage, laparoscopy, pregnancy

## Abstract

**Aim:**

Preterm birth is a worldwide health problem. After unsuccessful transvaginal cerclage, the transabdominal isthmo-cervical cerclage can be indicated. A laparoscopic approach has been described.

**Methods:**

A search was performed including the combination of: “((cerclage) AND (laparoscopy)) AND (pregnancy)”. A systematic review was performed to compare indications, outcomes, techniques, and safety.

**Results & discussion:**

42 articles were found through database search. 30 articles were included for review. By reviewing the literature, the transabdominal cervico-isthmic laparoscopic cerclage is highly effective in selected patients with a history of refractory cervical insufficiency. This technique has a high neonatal survival rate when placed in preconceptional or post conceptional patients. Moreover, laparoscopic cervical cerclage is a safe procedure when laparoscopic expertise is present.

## Plain language summary

A transvaginal cerclage is a procedure that places a stitch or tape within the cervix to mechanically close the cervix to prevent it from opening too early during pregnancy. However, if transvaginal cerclage fails, doctors may recommend a transabdominal isthmo-cervical cerclage using a laparoscopic approach. A systematic review analyzed 30 articles to compare the reasons for surgery, outcomes, techniques, and safety. The review suggests that this procedure is highly effective for certain patients with a history of refractory cervical insufficiency. It appears to be a safe technique with a high rate of survival for newborns and rare complications.

Every year, 15 million preterm deliveries are reported, and this number is rising according to the WHO. Across 184 countries, the rate of preterm babies ranges from 5% to 18% of live births [1]. Preterm birth, defined as a delivery before 37 weeks, is a leading cause of neonatal morbidities and mortality. Multiple pregnancies, infections (chorioamnionitis, urinary tract infection, etc.) and chronic conditions are common causes of preterm birth. However, cervical insufficiency may be a cause of preterm birth or second trimester and early third-trimester fetal loss. It is diagnosed after a history of painless, progressive shortening and dilatation of the cervix which usually happens in the second trimester. A transvaginal ultrasound with a cervical length measurement of less than 25 mm before 24 weeks of gestation may confirm the diagnosis. Historically, cervical insufficiency is treated by progesterone or cervical cerclage. Shirodkar, and then MacDonald described for the first time the techniques of transvaginal cervical cerclage [2]. Indications of the transabdominal route are failed transvaginal cerclage, trachelectomy, or absent vaginal cervix. With the development of minimally invasive surgery shown in the last decades, a new approach has been suggested for the transabdominal cerclage. The first laparoscopic cervical cerclage (LCC) was described by Lesser *et al.* [3] and Scibetta *et al.* in 1998 [4]. Since these publications, several manuscripts have been published to share every single institution's experience. This article aims to review the indications, outcomes, techniques, and safety of laparoscopic abdominal cerclage.

## Methods

### Strategy of research

The research was performed using the PubMed database. Filters applied: in the last 5 years. A search was performed including the combination of the following words: “((cerclage) AND (laparoscopy)) AND (pregnancy)”. The search and selection criteria were restricted to the English language.

### Selection of articles

**Figure 1.**
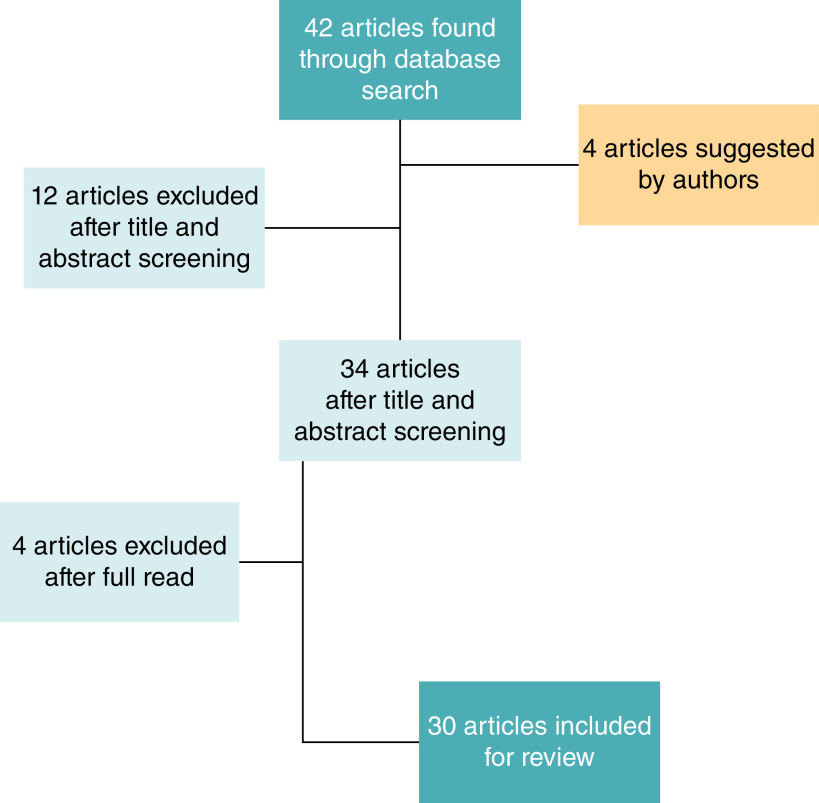
PRISMA chart.

The selection procedure followed the Preferred Reporting Items for Systematic Reviews and Meta-analysis (PRISMA) principles and is presented using a PRISMA flow chart (Figure 1). Recent articles were prioritized. Evidence included human, animal and cadaver data. Each article's title, abstract and text were reviewed for their appropriateness and their relevance. Full text analysis of eligible studies was performed. The initial list of selected papers was enriched by individual suggestions of the authors of the present review. Articles concerning robot-assisted transabdominal cerclage for the prevention of preterm birth were excluded. Reference lists from relevant papers were hand-searched for additional reports. The risk of bias and data synthesis was taken into consideration. Selective outcomes reporting bias was minimized by choosing the common outcomes in several studies. Confounding and publication bias was diminished by the adoption of standardized stricter inclusion and exclusion criteria.

### Extraction of data

The collection of data was done. Data were grouped depending on the type of clinical studies. Obstetrical outcomes and the differences in the surgical techniques were all reviewed.

Inclusion criteria were hospitalized high risk women who underwent a laparoscopic cerclage preconceptionally or post conceptionally.

The operative technique and characteristics were as follows:

- Gestational age at the moment of the cerclage.
- Type of the tape.
- Uterine manipulation.
- Surgical technique.
- Surgical complications.

The pregnancy outcomes observed were as follows:

- Mode of delivery.
- Gestational age at the delivery.

## Results

Thirty studies were included in our review. The PRISMA flow chart is presented in Figure 1. All these studies have reported the indications, surgical techniques, complications, and obstetrical and postnatal outcomes. The authors suggested four articles due to their relevant historical findings.

Lesser *et al.* [3] and Scibetta *et al.* [4] were the first authors who described the laparoscopic cervical cerclage. Since the publication of these two manuscripts, several case series have been reported in order to discuss the experience in their institution, the surgical and obstetrical outcomes and complications pre- and post-operative. The most common primary outcome was neonatal survival, and the secondary outcome was delivery ≥34 weeks of gestation. All the obstetrical outcomes are resumed in Table 1.

**Table 1.** Indications and outcomes of laparoscopic cervical cerclage across different included studies.

Objective	Study	Year	Method	Subjects	Results	Recommendations/conclusions	Ref.
Efficacy of LCC in twin pregnancy	Huang *et al.*	2019	Women with cervical incompetence associated with (i) one or more previous mid-trimester loss; (ii) a previously failed TVC; or (iii) a short cervix not amenable to TVC. All treated with prophylactic LCC in preconception (21) or post conception (3).	24	All delivered by cesarean section, with 16/24 of women delivering after 34 weeks and 21/24 women producing live births. In addition, conceived 4/24 following ovulation induction and 15/24 conceived with IVF	Prophylactic LCC might be beneficial in improving reproductive outcomes in high-risk infertile patients with known cervical incompetence who subsequently conceived twin gestations through IVF-ET treatment	[5]
Subsequent pregnancy outcomes when cerclage left *in situ* after LCC	Ades *et al.*	2019	women who were considered undergone a laparoscopic transabdominal cerclage and completed one pregnancy with the cerclage *in situ* became pregnant a second or third time.	22	In the first pregnancies, the neonatal survival rate was 22/22 and 19/22 of women delivered after 34 weeks. In the second pregnancies, the neonatal survival rate was 21/22 and 19/22 of women delivered after 34 weeks. In the third pregnancies, the neonatal survival rate was 3/3 and 3/3 women delivered after 34 weeks of gestation.	When left *in situ* for subsequent pregnancies, LCC is associated with a high rate of neonatal survival	[6]
Outcomes of pre-conception LCC	Saridogan *et al.*	2019	women at high risk of second trimester miscarriage and preterm delivery due to cervical insufficiency undergoing pre-conception LCC	54	Over 36 pregnancies progressing beyond the first trimester, the live birth rate was 33/36 The mid-trimester loss rate was 3/36 with delivery rates after 37 weeks of 27/36 and between 34–37 weeks of 3/36 and 23–34 weeks of 3/36.	LCC is feasible, safe and effective when TVC fails or is not possible.	[7]
Anatomical and functional changes in arteries of uterine circulation after modified laparoscopic transabdominal cerclage in pregnancy	Nemescu *et al.*	2020	Pregnant woman who underwent an LCC at 12 weeks of gestation, whose fetus developed growth retardation after 32 weeks.	1	Rich collateral vascularization develops within few weeks from the insertion of the cerclage. An adaptation of the circulation of Uterine Areteries that aims to ensure adequate flow to the placenta. A significant increase in cervical length during gestation.	Modified LTAC allows a safer technique, the impact of the cerclage on the pregnancy circulation and hemodynamics and fetal growth and development	[8]
Outcomes of LCC	Ades *et al.*	2018	pregnant women who underwent LCC in pregnancy, at 6–11 weeks gestation, from 2011 to 2017. Eligible women had cervical insufficiency and were not suitable for a TVC	19	The perinatal survival rate was 100%. There were no complications. The average gestational age at delivery was 37.1 weeks. 16/19 women delivered after 34 weeks.	LLC is a safe and effective procedure in women with poor obstetric histories. It requires the correct skill, expertise and patient selection.	[9]
Fertility outcomes after preconceptional laparoscopic abdominal cerclage for second-trimester pregnancy losses	Demirel *et al.*	2020	Patients who underwent LAC in the nonpregnant state for a second-trimester pregnancy loss despite the placement of vaginal cerclage between June 2012 and February 2020	40	No significant difference in spontaneous pregnancy rates before and after LCC, and in times to pregnancy before and after LCC	If placed during the preconceptional period, LCC does not delay achieving pregnancy and does not have a negative impact on the chances of conception.	[10]
Outcomes of the modified laparoscopic transabdominal cervical cerclage with transvaginal removing (MLTCC-TR)	Wang *et al.*	2020	Patients who underwent Preconceptional or postconceptional MLTCC-TR.	24	21/24 preconceptional LCC and 3/24 post conception. 27 live births. 22/27 were term infant	MLTCC-TR may be a relatively effective, feasible, and safe treatment for cervical insufficiency	[11]
Outcomes of LCC	Gremeau *et al.*	2018	LCC for women outside of pregnancy were performed, using the technique described by Dubuisson	25	21/25 pregnancies were recorded post cerclage including 5 early miscarriages and 16 cesarean deliveries The overall neonatal survival rate after cerclage was 76.2% vs 16.20% before surgery (p < 0.0001), with a 100% neonatal survival rate beyond the 1st trimester as compared with 21.6% before cerclage (p < 0.0001)	LCC seems to be safe, with less complication in comparison to abdominal cerclage	[12]
Outcomes of emergency LCC	Kavallaris *et al.*	2021	Patients underwent emergency LCC having cervical insufficiency with dilation in the second trimester due to extensive conization or re-conization and failed transvaginal cerclage	5	Average operation time 88 min, bleeding less than 100 cc, mean gestational age at surgery 14.4 weeks, mean gestational age by c-section was 38.1 weeks, survival rate 100%	Emergency LCC might be an alternative approach even in the early second trimester of pregnancy.	[13]
Outcomes of LCC	Ades *et al.*	2018	Women, who had a diagnosis of cervical insufficiency based on previous obstetric history and/or a short or absent cervix and were considered not suitable for a transvaginal cerclage, undergoing LCC	121	The perinatal survival rate was 98.5% with a mean gestational age at delivery of 35.2 weeks; 79.7% of babies were delivered at ≥34.0 weeks gestation.	Laparoscopic transabdominal cerclage is a safe and effective procedure resulting in favorable obstetric outcomes in women with a poor obstetric history.	[14]
Outcomes of surgical LCC “needle-free” technique	Abdel Azim *et al.*	2021	Patients who experienced previous transvaginal cerclage (TVC) failure or were not eligible for TVC, underwent pre- or postconceptional LCC using a needle-less mersilene tape	11	Median operation time of 62 min. no intra- or postoperative complications. 9/11 women became pregnant. No cases of miscarriage or mid-trimester loss.	Transabdominal laparoscopic “needle-free” cerclage is a safe and effective treatment option for a well-selected group of women at high risk of cervical incompetence.	[15]
Efficiency and safety of the new approach of laparoscopic cerclage	Shaltout *et al.*	2017	Women with history of two or more second trimester abortions or early preterm labor and having at least two previous unsuccessful vaginal cerclages	15	No complications during surgery. 12/15 delivered vaginally with the removal of cerclage, 2/15 had CS due to breech presentation and the cerclage was left in place and the last 1/15 has surgical evacuation	The new approach for laparoscopic cerclage is a safe, effective and reasonable treatment after failure of vaginal cerclage.	[16]
Cervical length after cerclage: comparison between laparoscopic and vaginal approach	Bolla *et al.*	2017	Comparaison between pregnant women undergoing a prophylactic LCC and those operated on TVC due to cervical insufficiency	38	18/38 LCC and 20/38 TVC. The cervical length in the TVC group showed a significant shortening while in the LCC group, the cervical length remained unchanged.	In patients with Cervical Insufficiency, LCC is associated with a better preservation of the cervical length throughout pregnancy as compared with TVC.	[17]
Efficiency of Modified LCC	Seo *et al.*	2019	Modified LCC performed to pregnent woman with cervical insuffiency	1	Doppler findings showed normal feto-placental circulation before and after the procedure. baby showed normal growth.	Modified laparoscopic cervicoisthmic cerclage is suggested as one of the treatment methods for pregnant women with refractory cervical incompetence.	[18]
Modified Laparoscopic Transabdominal Cervicoisthmic Cerclage	Chung *et al.*	2021	Pregnant women with history of recurrent pregnancy loss, who underwent a modified LCC	299	The surgery was performed at a mean gestational age of 12.5 weeks. There were 176/205 successful deliveries via cesarean section	Modified LTCC is a safe and feasible surgical option during pregnancy for patients with a history of RPL due to cervical factors.	[19]
Outcomes of LCC	Clark *et al.*	2020	124 Preconceptional women and 13 pregnant women, with a history of recurrent pregnancy loss, who underwent LCC	137	Neonatal survival rate was 93.8%, with mean gestational age at delivery 36.9	Laparoscopic cerclage is a safe, effective and reasonable treatment after failure of vaginal cerclage.	[20]

LCC: Laparoscopic cervical cerclage; MLTCC-TR: Modified laparoscopic transabdominal cervical cerclage with transvaginal removing; TVC: Transvaginal cerclage.

### Time of the procedure & outcomes

Ades *et al.* had an important experience in laparoscopic cervical cerclage. They shared the outcomes of 121 pregnancies after a pre-pregnancy laparoscopic cervical cerclage in women at high risk for pre-term from 2007 to 2017 [14]. The perinatal survival rate was 98.5% with a mean gestational age at delivery of 35.2 weeks. Moreover, Ades *et al.* published a paper concerning their experience in laparoscopic transabdominal cerclage in 19 pregnant women at 6–11 weeks of gestation [9]. The perinatal survival rate was 100% with an average gestational age at delivery of 37.1 weeks. Furthermore, Ades *et al.* reported the obstetric outcomes of subsequent pregnancies in women who had a laparoscopic transabdominal cerclage left *in situ* [6]. Of 22 women, the neonatal survival rate was 95% in the **s**econd pregnancy and 86% of women delivered after 34 weeks of gestation. On the other hand, the neonatal survival rate was 100% (3/3) in the third pregnancies, and 100% (3/3) of women delivered after 34 weeks of gestation.

### Modified LCC

The largest case series was reported by Chung *et al.* who underwent a retrospective observational cohort study of patients operated on modified laparoscopic transabdominal cervical cerclage from 2003 to 2018 with a sample size of 299 pregnant women with a mean gestational age of 12.5 weeks [19]. There were 176 of 205 successful deliveries via cesarean section with a fetal survival rate of 85.9%. This technique is described first by Shin *et al.* in 2015, and it differs from the conventional laparoscopic cervical cerclage by the origin of the insertion of the tape – laterally to uterine vessels and above the uterosacral ligament. The different surgical techniques are detailed in Table 2.

**Table 2.** Description of the surgical technique used across different included studies.

Technique	Year	Time of surgery	Uterine manipulator	Tape	Comment	Insertion and techniques	Knot	Complication/bleeding	Delivery/term	Ref.
Scibetta *et al.*	1998	Pre-conception	dilator 8 mm	Mersilene tape	Needle removed from each side; bladder carried out	Medial to uterine artery, posterior to anterior	Endoclose / anterior knot	No complications Minimal bleeding	Cesarean section / at term	[4]
Lesser *et al.*	1998	11 weeks	None	Mersilene tape	–	Medial to the uterine vessels at the level of the uterosacral ligaments	Anterior flat square knot	Bleeding of one uterine artery/controlled by clip and packing	Cesarean section/35 weeks	[3]
Gibb *et al.*	2016	Pre/post-conceptional	Manipulator if not pregnant	Mersilene tape	Bladder reflection is not carried out except in previous cesarean section	The needle is passed bilaterally between the uterine vessels and the cervicoisthmic junction, coming out through the posterior leaf of the broad ligament, approximately 1 cm above the uterosacral ligament	Posterior knot	–	–	[7]
Shin *et al.* Modified LCC	2015	12 weeks	None	polyfilament polyester (Dagrofil)	–	Posterior-to-anterior, laterally to the uterine vessels	Anterior knot	No complications/less than 100 cc	Cesarean section at 36 + 3 weeks	[21]
Ades *et al.*	2018	6–11 weeks	Uterine manipulator if non pregnant	No. 1 Prolene	In the non-pregnant cerclage, suture passed twice around the cervix	medial to the uterine vessels: anterior to posterior on the left and then posterior to anterior on the right side.	Anterior knot if pregnant	No complications	Cesarean section at 37.1 weeks	[9]
Whittle *et al.*	2009	Pre/post-conceptional	uterine manipuator if gravid; for the pregnant patient, a sponge on ring forceps was placed into the vaginal fornix	No. 1 Prolene	–	Posterior to anterior method, inserted bilaterally medial and posterior to the uterine vessels	Posterior knot	7 conversions to laparotomy due to uterine vessel bleeding or impaired surgical visibility; 2 pregnancies were lost perioperatively, 6 loss of pregnancy in the second trimester due to the consequences of acute or subacute chorioamnionitis.	35.8 weeks	[22]
Wang *et al.* Modified LCC with trans-vaginal removing	2019	Pre-conception or post conception (10.9 weeks)	–	Mersilene tape	–	Transvaginal Puncture at 4 o'clock position of posterior fornix, Transvaginal Penetration of Endopath Ultra Veress Insufflation Needle punctured at 7 o'clock position after surrounding cervical isthmus to slip the tape into Vagina, Transvaginal knotting and fixiation	Vaginal knot	No severe perioperative complications	80.7% vaginal delivery, 37.2 weeks if pre-conception, Term delivery if post conception	[11]
Kavallaris *et al.*	2021	14.4 weeks Emergent LCC, dilated cervix	No manipulator	Mersilene tape	Bladder pushed downward	Posterior-to-anterior direction and bluntly placed through the paracervical tissue under the transvaginal ultrasound view	intra-corporal knot in the ventral cervicoisthmic segment.	Bleeding less than 100 cc / no complications	Elective cesarean section at 38 weeks	[13]
Shaltout *et al.*	2016	Pre-conception	No manipulator	Mersilene tape	Bladder dissected downward	medial to uterine vessels bilaterally from anterior to posterior When the needles' blunt ends pierce the vaginal vault, the assistant pull them through the posterior vaginal fornix.	Intravaginal knot inserted behind the of the cervix	No complications / no bleeding	12/15 vaginal delivery at 36.3 weeks	[16]
Petry *et al.*	2020	Pre-conception	dilator 8 mm	Mersilene tape	Bladder dissected downward	Medial to uterine artery	No knot, tape is fixed by Protack			[23]
Gremeau *et al.*	2018	Pre-conception	Dilator 6 mm	Mersilene Tape	No displacing of bladder	Posterior to anterior, medial to uterine artery	Anterior knot	Minor hemorrage	The overall neonatal survival rate was 76.2%	[12]
Vissers *et al.*	2017	Gravid at 11–14 weeks	McCartney tube	Mersilene tape	Bladder dissected downward	Anterior to posterior, medial to uterine artery	Posterior knot	No complication	–	[24]

LCC: Laparoscopic cervical cerclage.

### LCC in twin pregnancy

However, there are few articles introducing the efficiency of laparoscopic cervical cerclage in twin pregnancy. The largest sample of twin pregnancy undergoing laparoscopic cervical cerclage was studied by Huang *et al.* in 2019: 24 women delivered by cesarean section, with 16/24 after 34 weeks and 21/24 women producing live births [5].

### Emergency LCC

However, the outcomes of emergency laparoscopic cervical cerclage were introduced in one case series of 5 patients by Kavallaris *et al.* in 2021 [13]. Patients, who underwent cervical cerclage in a mean gestational age of 14.4 weeks due to cervical insufficiency and dilation, delivered by cesarean section at 38.1 weeks with a 100% survival rate.

Moawad *et al.* thoroughly studied the safety and beneficence of laparoscopic transabdominal cervical cerclage in their systematic review with meta-analysis [25], where they compared 25 studies (1116 patients) on transabdominal cerclage placed by laparotomy to 15 studies (728 patients) on transabdominal cerclage performed by laparoscopy. They reported a higher neonatal survival rate in the laparoscopic group without a difference in peroperative complications between the two approaches. The time of surgery was longer in laparoscopic cases, but the length of stay in the hospital was significantly shorter.

## Discussion

Laparoscopic cervical cerclage is a surgical approach suggested to prevent premature labor in high-risk women with a history of failed transvaginal cerclage, trachelectomy, or absent vaginal cervix. Hulshoff *et al.* [26] compared indirectly the laparotomy to the laparoscopic approach in transabdominal cerclage. They concluded that there is no superiority of one technique over the other one. On the other hand, Marchand *et al.* [27] showed that LCC seems to be safe in comparison with the transabdominal approach in the management of cervical insufficiency with a statistically significant lower incidence of fetal loss, blood loss, and rate of hemorrhage. In our case, this systematic review evaluates the different indications and surgical techniques of LCC, and obstetrical outcomes without any direct comparison. The summary of results and comparison with other literature are discussed as follows:

### Indications of laparoscopic cervical cerclage

A select cohort of women, with a history of second-trimester abortions or early preterm labor due to cervical insufficiency, should benefit from periconceptional counseling for laparoscopic cervical cerclage [28]. These women must have previous unsuccessful vaginal cerclage or failure of vaginal insertion of cerclage because of congenitally short cervix, cervical conization, or cervical scarring. The minimal number of second trimester abortions, or the number of previous unsuccessful transvaginal cerclage considered in the inclusion criteria are not identical in several reviewed case series. Although clear recommendations concerning abdominal cerclage are provided, no evidence is proven as to the preferred approach between laparotomy, laparoscopy, and the vaginal route. The MAVRIC study [29] is a multicenter randomized controlled trial where patients were assigned randomly (1:1:1) to receive transabdominal cerclage, high vaginal cerclage, or low vaginal cerclage either before conception or before 14 weeks of gestation. The objective is to compare transabdominal cerclage or high vaginal cerclage with low vaginal cerclage in women with a history of failed cerclage. It showed that abdominal cerclage prior or at the beginning of pregnancy reduces the rate of late miscarriage and premature delivery compared with vaginal cerclage. In addition, Moawad *et al.* compared in their meta-analysis 25 studies (1116 patients) on transabdominal cerclage placed by laparotomy to 15 studies (728 patients) on transabdominal cerclage performed by laparoscopy and they reported a higher neonatal survival rate in the laparoscopic group without difference in peroperative complications [25].

### Time of the surgery: before or after conception

The laparoscopic cervical cerclage can be provided before conception or after conception, most commonly during the first trimester of pregnancy. There is no cohort study dealing with the difference between the preconceptional and postconceptional laparoscopic cervical cerclage. A systematic review undergone by Tulandi *et al.* [30], evaluated the efficacy of abdominal cerclage via laparoscopy before versus after conception, and did not find any significant difference in the live birth rates when abdominal cerclage was performed before or during pregnancy. Although no difference in obstetrical primary outcomes is shown between the two groups, there is a risk of perioperative pregnancy loss among gravid women reported in some manuscripts. Whittle *et al.* [22] studied the effect of timing for cerclage on pregnancy outcome in their case series. The mean gestational age for delivery was 32.9 weeks and 34.5 weeks when cerclage was placed in gravid and non-gravid women respectively. Additionally, they reported seven pregnancy failures when cerclage was placed in pregnancy, while two failures were noted when the cerclage was done on non-gravid women. Of concern, they concluded that the timing of cerclage placement did not influence the gestational age at delivery, but cerclage failure did occur more often when the cerclage was placed during that pregnancy. However, multiple anatomical changes that occurred during the first trimester of pregnancy should be considered during the surgery and can modify the surgical technique. The difficulty of uterine manipulation, developing the uterine windows, and the uterine vessels engorgement among gravid women can affect the uterine artery skeletonization and the placement of the knot during the operation. Of note, the conversion to laparotomy did occur more frequently when the patient was pregnant in Whittle *et al.* case series. On the other hand, the disadvantage of preconception cerclage is that pregnancy may either never occur or result in an early loss unrelated to cervical incompetence.

### Surgical technique

#### Uterine manipulation

In the case of gravid women, using a uterine manipulator during laparoscopic cervical cerclage is not possible. In the modified LCC, four trocars were used with atraumatic forceps for uterine manipulation [21]. Sponge on ring forceps placed into the vaginal fornix was used for pregnant women in Whipple *et al.* experience [22]. In preconceptional laparoscopic cerclage, tenaculum and dilator of 6 or 8 mm or a transcervical uterine manipulator are commonly used during this surgery [4].

#### Type of the tape

Two types of tapes are described in the reviewed studies: The conventional Mersilene Tape and Prolene suture. Whittle *et al.* [22] were the first who decided to use Prolene instead of Mersilene, inspired by the choice of Rust *et al.* [31] in choosing of suture material for a vaginal cerclage. Ades *et al.* used Prolene n 1 in their laparoscopic cerclage (5–7). Shin *et al.* [21] used Mersilene tape in their case series of postconceptional laparoscopic cerclage, and had an average gestational age at delivery of 36.2 weeks with a fetal survival rate of 95% without reported complications. On the other hand, we found a case series by Whittle *et al.* [22] where Prolene n°1 was used, six cases of laparotomy conversion, and an average gestational age at delivery of 32.9 ± 8.8 weeks. However, Ades *et al.* [9] used Prolene in 19 gravid women before 11 weeks, and found that all patients delivered with an average gestational age of 37.1 weeks without any reported complications. Of concern, no cohort study dealing with the comparison between the two types of tape was found in the literature. However, Mersilene tape is more likely resistant to uterine contractions during labor, but it causes fibrosis around and within the braided fibers. Thus, Prolene known as mono-filament non-braided suture with minimal tissue reactivity and durability, are easier to insert and remove, but it may be more likely to cut through the uterine tissue.

#### Method of tape insertion

Traditional laparoscopic cervical cerclage is based on a three-port laparoscopic approach with a fourth suprapubic assistant port. Four ports are preferable in gravid women to facilitate uterine manipulation. An incision is performed at the level of the utero-vesical fold in the visceral peritoneum and extended laterally to the broad ligaments. It is not necessary to carry out the bladder reflection systematically, while it is preferable in the case of previous cesarean sections. Most surgeons dissected the uterovesical and paravesical spaces and made a broad ligament window after identification of the ureters and uterine arteries. Although the suture may be inserted in either direction, there was no evidence that placing the suture from anterior to posterior has advantages more than the opposite direction. Some authors preferred the anterior to posterior direction for better visualization, and reduced risk of bowel injury and bladder erosions. Straight or straightened needles can be used during this procedure because they presented a more accurate direction of the suture. Furthermore, the suture is then passed at the level of the uterine isthmus medial to the uterine vessels. However, the tape is inserted laterally to the uterine vessels and above the ureters at the level of the uterine isthmus, above the uterosacral ligament in the modified laparoscopic cervical cerclage [21]. This technique reduced the operation time and blood loss and improved the recovery time in comparison with a traditional laparoscopic cervical cerclage. This modified method did not completely block blood flow to the uterus, and the development of existing collateral circulation did not affect fetal growth. However, Shaltout *et al.* [16] described their technique where after insertion of the needle bilaterally with an anterior to posterior direction, both needles were passed through the cervical tissue medial to uterosacral ligaments toward the posterior vaginal fornix, and the tape was tied behind the intravaginal segment of the cervix. On the other hand, Wang *et al.* [11] also described a different technique where a Transvaginal Mersilene Needle was inserted at the 4 o'clock position of the posterior fornix until its tip appeared down the vesico-uterine peritoneum, then a transvaginal penetration of the Endopath Ultra Veress insufflation needle was punctured at 7 o'clock position to slip the tape into the vagina after surrounding the cervical isthmus, to finish with transvaginal knotting.

#### Location of the knot & cerclage removal

Three different locations of the knot were described: anterior, posterior, and intravaginal knot. Anterior knots have the advantage of avoiding the risk of adhesions in the Douglas pouch, and can be easily removed in laparoscopy, but may increase the risk of erosion into the bladder. On the other hand, posterior knots can be removed via posterior colpotomy in case of pregnancy failure in the second trimester and this allows vaginal delivery. To avoid the unindicated cesarean section at term for removing the intracorporal cerclage knots, some authors described the intravaginal knot method to simplify knot removal [16]. To sum up, the knot is preferably placed anteriorly in post conceptional cerclage to simplify the difficulty in accessing the posterior cul de sac, while the posterior knot is preferable in preconceptional cerclage. To simplify the cerclage removal, Ades *et al.* [6] conducted a case series describing the outcomes of subsequent pregnancies when the laparoscopic cervical cerclage was left *in* *situ* and concluded a high neonatal survival rate after 34 weeks of gestation even in third pregnancies.

### Safety & complications

Multiple complications are described by reviewing the literature. Some complications were related to the laparoscopic surgery and others to the transabdominal cervical cerclage procedure. Although Moawad *et al.* [25] reported a rate of 1% of peroperative complications in their meta-analysis, some reports described a higher complication rate. Specific complications such as bleeding from uterine vessels and loss of pregnancy when cerclage was performed in gravid women are the most reported. The overall complications rate reported was 1.3% in the Ades *et al.* case series of 121 interval cerclage [14]. There was one post-operative wound infection, and one intra-operative bladder injury and one uterine fundal perforation, both laparoscopically sutured. On the other hand, the case series of Whittle *et al.* [22] was the first largest study that reported a serious rate of complications, especially in the non-interval procedure. It included 31 cerclages during pregnancy and 34 preconceptional cerclage, there were two fetal losses (2/31) and seven conversions to laparotomy (7/65) due to bleeding from the uterine vessels (5), or impaired visibility (2). Six of the seven patients who required conversion to laparotomy were pregnant. Ades *et al.* [9] did not report any serious complications in their case series where cerclage was placed in 19 pregnant women before 11 weeks of gestation. Chung *et al.* [19] published recently the outcomes of the modified laparoscopic cervical cerclage when placed in gravid women with a mean gestational age of 12.5 weeks with the largest sample size (299). The operative complications rate was 0%. To sum up, we can consider that the operative complications seem uncommon in these procedures and depend directly on the operators' skills and expertise.

### Obstetrical outcomes

Moawad *et al.* [25] reported in their meta-analysis improved obstetric outcomes associated with the laparoscopic approach in comparison with laparotomy and robotic surgery. They noted an overall neonatal survival rate of 89.9% and 96.5% when first trimester pregnancy losses were excluded, with 82.9% deliveries after 34 weeks. Moreover, over the 121 pregnancies included in Ades *et al.* case series [14], where the cerclage was provided in preconception, we report a perinatal survival rate of viable pregnancies of 98.5% with an average gestational age at delivery of 35.2 weeks. 79.7% of these patients delivered after 34 weeks of gestation. On the other hand, over the 205 pregnancies followed up after their post conceptional modified laparoscopic cerclage in Chung *et al.* study, 176 (85.9%) delivered successfully live births. 60 of 205 patients (29.2%) delivered before 37 weeks [19]. Furthermore, the neonatal survival rate was 93.8%, with mean gestational age at delivery 36.9 in Clark *et al.* study [20], where 124 preconceptional women and 13 pregnant women, with a history of recurrent pregnancy loss underwent laparoscopic cervical cerclage.

### Strengths & limitations

To our knowledge, this is a large and new systematic review exploring the surgical technique of the laparoscopic approach of abdominal cerclage with obstetrical outcomes. On the other hand, the small sample size in some of the included studies, their retrospective design, and the lack of standardized criteria for the technique, and timing of the operation represent the major limitations of this systematic review, thus making it difficult to conclude any convincing evidence on the management strategies. We also included case reports and case series, thus facing a higher risk of publication bias and decreasing the level of the evidence of our findings. Moreover, indications for delivery are not discussed in any study: patients with an indicated preterm labor (for placental disease for example) should be excluded.

## Future work

Future randomized cohort studies with a larger sample size are required to evaluate the best technique (medial or lateral to the uterine artery, posterior-to-anterior direction or the opposite), the most efficient type of tape (Mersilene or Prolene), and the best timing for this procedure (preconception or first trimester) to have better obstetrical outcomes. However, these studies need significant statistical power. Furthermore, the beneficial outcomes of laparoscopic cervical cerclage may be a reason for medical laboratories and manufacturers to invest more in the production of resorbable tape based on native tissue. Moreover, further studies are needed to evaluate the efficiency of laparoscopic cervical cerclage in multiple pregnancies.

## Conclusion

Although the United Kingdom National Institute for Health and Care Excellence (NICE) classified laparoscopic cerclage as a procedure with limited evidence for success, multiple studies showed that transabdominal cervico-isthmic laparoscopic cerclage is highly effective in selected patients with a history of refractory cervical insufficiency. This technique has a high neonatal survival rate when placed in preconceptional or post conceptional patients. Moreover, laparoscopic cervical cerclage seems to be a safe procedure when the correct skill and laparoscopic expertise are present.

**Summary points**

- After unsuccessful transvaginal cerclage, the transabdominal isthmo-cervical cerclage can be indicated in a selected high-risk patient. A laparoscopic approach has been described.
- The transabdominal cervico-isthmic laparoscopic cerclage is highly effective in selected patients with a history of refractory cervical insufficiency.
- This technique has a high neonatal survival rate when placed in preconceptional or post conceptional patients.
- Laparoscopic cervical cerclage is a safe procedure when laparoscopic expertise is present.
